# Evaluation of Physical Characteristics and Sorption of Cement Mortars with Recycled Ceramic Aggregate

**DOI:** 10.3390/ma14247852

**Published:** 2021-12-18

**Authors:** Agata Stolarska, Teresa Rucińska

**Affiliations:** Faculty of Civil and Environmental Engineering, Department of Building Physics and Building Materials, West Pomeranian University of Technology in Szczecin, al. Piastów 50A, 70-311 Szczecin, Poland; Teresa.Rucinska@zut.edu.pl

**Keywords:** sorption, sorption isotherm, cement mortar, recycling, recycled ceramic waste

## Abstract

The subjects of this study were mortars with varying amounts of recycled ceramic aggregate (RCA). As part of the fine aggregate, the RCA volume share is 10%, 20%, 30%, 50% and 100%. First, fresh mixture parameters were evaluated, such as consistency and air content measurement by pressure method. Next, specimens were molded for compressive strength and flexural strength tests after 7, 28 and 56 days of curing. The thermo-humidity parameters of the composites, i.e., coefficient of capillary action and thermal conductivity coefficient were also investigated using nonstationary method. Sorption kinetics of the mortars at different moisture conditions at 20 °C were also evaluated. Sorption tests were carried out using two methods: TM and DVS. The sorption isotherms were plotted on the basis of equilibrium moisture content for the materials tested. The isotherms obtained by the two methods were evaluated. The results allowed us to draw conclusions on the physical and mechanical parameters of the composites with different amounts of RCA and to evaluate the ability to absorb moisture from the environment by these types of materials. A clear decrease in the compressive strength after 28 days of curing compared to the reference mortar was recorded after using 30% to 100% of RCA (approx. 26% to approx. 39%). Changes in flexural strength were significantly smaller, reaching no more than approx. 7.5%. It was shown that the amount of RCA translates into the ability to sorb moisture, which may affect the application of this type of composites. The amount of RCA translates also into the thermal conductivity coefficient, which decreased with increasing amount of RCA.

## 1. Introduction

The increasing amount of construction wastes are a clear environmental threat. Therefore, the management of these resources is one of the greatest challenges of the modern world. Researchers from all over the world continuously look for new ways to utilize the raw or processed waste materials for different industrial processes [1,2,3,4,5]. Significant improvements can be found in the technology of construction materials, mainly in cement composites such as mortars and concretes [6,7,8,9,10]. Recycled materials and waste materials in the form of powders are used as cement replacement or filler, while crushed materials can replace natural fine and coarse aggregates [11,12,13,14].

The authors of [15] have conducted a review of publications presenting the results of studies on the physical, mechanical and microstructural characteristics of aggregates obtained from ceramic wastes produced from different structures and products (e.g., bricks, tiles, etc.). Based on the results of the study, it was concluded that concrete with ceramic aggregate has comparable technical properties to ordinary concrete. The results have shown that the compressive strength, permeability, adhesion, etc. of concrete with ceramic aggregate meets the required criteria set by various international standards and technical requirements, which confirms the suitability of ceramic waste as an effective substitute for natural aggregates in structural concrete. However, the authors have observed that the mechanical performance of reinforced concrete elements produced with ceramic waste has not been studied and propose this topic for future research.

The topic of using recycled materials is often addressed in the literature. For example, the authors [16] have provided a comprehensive literature review on the possibility of using recycled construction and demolition waste in the production of composites. They have focused on investigating the use of recycled wood, paper, cardboard, metal, glass, mineral wool, gypsum, concrete and ceramics as raw materials for composite materials. Composites offer the possibility of using and combining recycled materials into products with new properties. In the paper [16], the possibilities of using easily obtained demolition wastes for production of composites were evaluated. The composition, contamination, degradation and recycling of construction materials were discussed to elucidate potential challenges for composite materials manufacturers. One of the conclusions drawn is that the use of recycled materials as matrix, filler or fiber results in materials with weaker mechanical properties when compared to raw materials. It has been reported that in the case of polymer matrix, degradation occurs under the influence of high temperature and moisture absorption. In the case of recycled wood fibers, the authors have reported that the hydrophilic character of cellulosic fibers has a negative effect on the fiber adhesion in a hydrophobic plastic matrix, which often leads to lowered mechanical performance.

The authors [17] have investigated the use of ceramic waste as aggregate replacement in cement concrete. For the study, ceramic waste was used as fine and coarse aggregate with different replacement rates of 0%, 10%, 20%, 30%, 40% and 50%. The authors determined the properties of fresh and hardened concrete. Results of their study have shown that the ceramic wastes is suitable if its content does not exceed 20%. For this replacement level, produced concretes achieved comparable results as the reference ordinary concrete.

Other researchers [18] in their review paper focused on the influence of ceramic waste on the physical, chemical, mechanical and durability properties of different forms of concrete. Higher mechanical performance and durability have been reported for concrete containing ceramic waste as a substitute for cement and aggregate. It was also concluded that the use of ceramic waste in construction materials not only reduces the production costs but also increases the sustainability. The authors of [19] also carried out an experimental study of self-compacting concretes (SSCs) using ceramic waste powder from bone china cup plate-type ceramic. In the produced mixes, the sand was replaced by weight with ceramic waste powder at 0%, 10%, 20%, 30%, 40% and 50% rates. Similar to authors [17], high SCC performance were obtained for ceramic waste powder content of 20%.

In work [20], the authors used granulated ceramic waste (<0.125 mm) as a filler in self-compacting concrete (SCC) with 550 kg/m^3^ of cement. The fresh and hardened properties were determined for concretes with replacement of %weight cement in the amount of 5%, 10%, 15% and 20%. As a result, it was found that the use of ceramic filler had a positive effect on the viscosity of the mixtures, but there was a slight decrease in the strength values. Thus, it was concluded that finely ground ceramic waste can be added up to 15% if concrete mix properties and strength are evaluated together.

In work [21], researchers performed an evaluation of the characteristics of waste ceramic powder including chemical composition, morphology and activity. The authors produced concretes with three different strength class (25 MPa, 50 MPa and 75 MPa), where waste ceramic powder was used as a partial cement replacement. The proportion of waste ceramic powder varied between 10%, 20%, 30% and 40%. The researchers conducted a number of tests of fresh and hardened concrete, including the durability of the composite. The results showed that, depending on the replacement rate, the performance of concrete changed. To optimize the rheological and mechanical characteristics of concretes with ceramic waste powder, the authors developed a performance index (PI). The PI considers workability, compressive strength and durability as performance measures in selecting the most appropriate level of cement replacement with ceramic waste powder. They also suggested that PI could be expanded to include additional criteria. In this approach, cement can be replaced by up to 40% with ceramic waste powder.

The authors of [22] used ceramic polishing waste, which is generated during polishing of ceramics, as a substitute for cement paste. The researchers made a number of mortar mixes with different cement, water and ceramic polishing waste contents. The study evaluated the workability of the mixes, as well as the strength. The authors also determined the microstructure using SEM. It was found that by adding up to 20% by volume of ceramic polishing waste, it was possible to reduce the amounts of cement in paste by 33%, with simultaneous increase in the compressive strength of at least 85%. Results of microstructure evaluation showed an increase in compaction.

Other studies [23,24] have focused mainly on the mechanical performance of composites containing recycled wastes. The issues related to moisture transport in those materials have been partially addressed. One study [24] evaluated the application of ceramic wastes in ecological concrete and precast concrete. The elements were mainly studied in terms of their compressive strength. Similarly, in [25], the use of recycled aggregates from construction and demolition waste to produce various precast concrete elements was studied. Tests have been also conducted to determine the durability and permeability of studied specimen.

In a study [26], five mixes were produced with a w/c = 0.40 and different recycled aggregate contents. The first group of mixes had 10%, 20% and 30% replacement of coarse aggregate by volume, while the second 10% and 20% replacement of both fine and coarse aggregate. Mixes with only coarse aggregate performed better than the mixed aggregate mixtures in terms of flexural and tensile strength, shrinkage, absorbability and chloride ion permeability rate. The sorption of the samples was also determined. This characteristic can be used as an indication of the durability of concrete and is related to the self-curing properties. An increase of sorption with the increase of recycled ceramic aggregate (RCA) content was observed. It shows that the mix with 30% RCA had the highest sorption among studied mixes. A significant increase of secondary sorption rate was observed when the content of coarse RCA aggregate increased from 20% to 30%. This was also present in a study [27] when RCA replacement was increased from 25% to 50%. Another conclusion drawn in [27] was that recycled concretes have worse mechanical properties and higher sorptivity than ordinary concrete. It has been observed that majority of physical and mechanical properties of hardened concrete decrease with the increase of recycled aggregate content.

The results presented in [26] show that the initial sorptivity of the mixes with 10% and 20% replacement rate of both fine and coarse aggregate was slightly higher than for mixes with only coarse aggregate replaced. Study [26] showed that this results from increased mortar content caused by smaller aggregate size compared to mixes with only the coarse aggregate replaced. Increase in the mortar volume for mixes with RCA is reflected in the secondary sorption results. Researchers [26] have concluded that the pore dispersion in the structure caused by higher volume of paste in mixes with mixed RCA influences the sorptivity. They have also found that with increased volume of RCA, there is a greater reduction in secondary sorption compared to the initial results. This was also due to the fact that the pore structure of RCA has a greater ability to absorb additional water than natural aggregates.

The sorption has been also tested in [28] to evaluate the durability of concrete made from recycled aggregate. Three concrete mixes containing 0%, 50% and 100% recycled aggregate were tested. The specimens were tested after 3, 7, 28 and 56 days of curing. The quality of concrete near the surface, according to [28], depends on its curing, which affects the rate at which the sample will absorb water. The water absorption was tested in a single direction. It was shown that the water sorptivity decreased with the increase of curing time for a given recycled aggregate content in the mix.

The authors of [29] showed that the water absorption increased up to 34% when the natural aggregates were replaced up to 50%, and the percentage of replacement did not affect the compressive strength of concrete, which is only affected by the quality of recycled aggregate used. However, in work [29], the replacement of fine aggregated significantly more influenced negatively the water absorption of concretes than replacement of coarse aggregate.

Based on the review of literature presented above, the authors planned the experiment on cement mortars with the addition of ceramic recycled waste. The results presented in this paper supplement the knowledge on the use of recycled ceramic waste in cement composites. Apart from the determination of basic mechanical properties, an attempt was made to evaluate the sorptivity of cement mortars with porous waste aggregate obtained from ceramic rubble. Two measurement methods were used to determine the sorptivity of mortars, namely the traditional gravimetric method [ISO 12571] and the DVS method. Understanding the adsorption properties of the mortar with recycled ceramic aggregate will allow us to use the obtained information to simulate the hygrothermal processes. Material sorption isotherms are one of the parameters necessary to describe materials, e.g., in the WUFI program. This will increase the accuracy of calculations for the new material, which is a mortar with recycled ceramic aggregate.

## 2. Materials and Methods

### 2.1. Materials

#### 2.1.1. Sand and Waste Ceramics Aggregate

**Figure 1:**
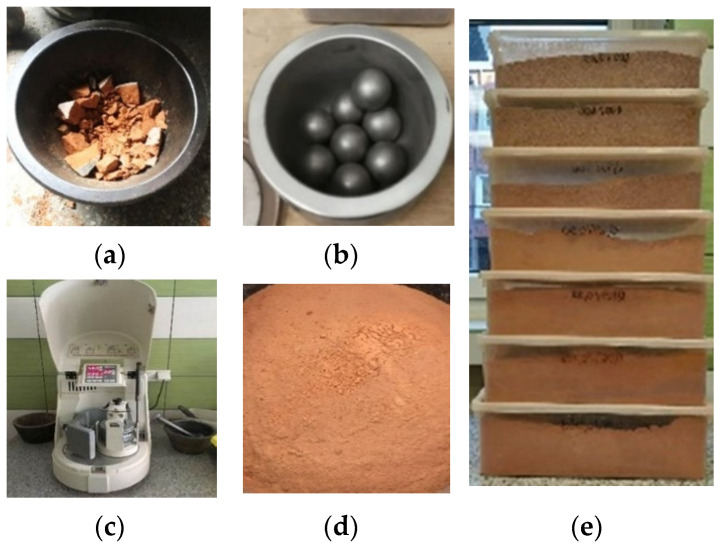
Set for grinding and crushing of ceramic rubble; (**a**) premilling ceramic waste; (**b**) grinding balls a diameter of 20 mm (hardened, stainless steel—Fe-Cr); (**c**) ball mill; (**d**) ground ceramic rubble; (**e**) waste aggregate divided into fractions.

**Figure 2:**
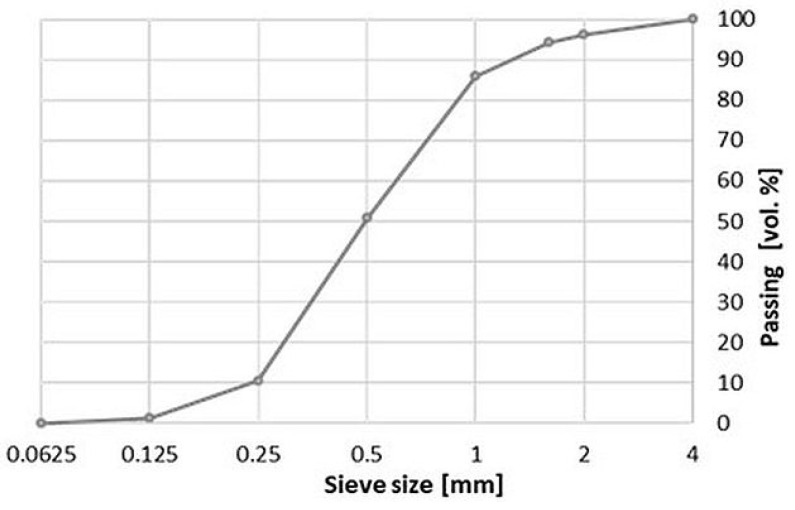
The grading of sand and waste ceramic aggregate after crushing into fractions.

Natural fine aggregate (quartz sand obtained from Bielinek, Poland) and waste aggregate obtained from recycled bricks, building blocks and demolition tiles were used to make mortars. It was assumed that the waste aggregate will be a substitute for the natural aggregate in the amount of 10%, 20%, 30%, 50% and 100% by volume, due to the different volume density of both aggregates. The grading of ceramic waste aggregate was made to compel with the grading of natural sand. A ball mill (the Planetary Mono Mill PULVERISETTE 6 classic line, Retsch GmbH, Idar-Oberstein, Germany) was used to grind the ceramic rubble (Figure 1). Next, the milled material was passed through standard sieves, and the grading was adjusted (Figure 2).

#### 2.1.2. Cement and Water

Portland cement CEM I 42.5R (Górażdże, Poland) according to EN 197-1 classification, with compressive strength of at least 42.5 MPa, was used for production of mortars. The cement has high early strength R (after 2 days of maturing) and standard strength after 28 days of maturing, stable quality and high heat of hydration. Tap water from the water supply system was used to make the mixtures. According to EN 1008, drinking water as mixing water does not require additional testing.

### 2.2. Mix Preparation

In this study, six cement mortars were produced: one reference mortar (MR) with a composition corresponding to a standard cement mortar (cement-to-sand weight ratio 1:3, W/C = 0.5) and 5 mortars with the addition of recycled ceramic waste as a volume replacement for natural aggregate in the amounts of 10%, 20%, 30% 50% and 100% (M10, M20, M30, M50 and M100, respectively).

The density of ceramic waste aggregate (A_WC_) was determined as 2.69 g/cm^3^. CEM I 42.5R cement has a density of 3.11 g/cm^3^ (PC), while sand has a density of 2.65 g/cm^3^ (A_NS_). The density of the materials was determined by the pycnometric method according to EN 1097-1: 2001. The particle size distribution of the test sample was fraction 0/0.63. The average of these measurements was taken as the final result.

Compositions of mixtures are presented below:

- MR—reference mix, 100% A_NS_
- M10—mix with 10% A_WC_ + 90% A_NS_
- M20—mix with 20% A_WC_ + 80% A_NS_
- M30—mix with 30% A_WC_ + 70% A_NS_
- M50—mix with 50% A_WC_ + 50% A_NS_
- M100—mix with 100% A_WC_ + 0% A_NS_

The ingredients of the mortar were combined using a mixer. The dry ingredients were placed in the mixer and mixed for 3 min to ensure a uniform blend. The water for mixing was added gradually while mixing in an amount resulting in W/C = 0.5. Subsequently, additional water was gradually added while mixing the mortar components with the ceramic aggregate to obtain a consistency comparable to the reference mixture. It was necessary due to the rapidly progressing absorption of the mixing water by the ceramic grains. The mixing of all ingredients was continued for another 3 min. After the components were mixed, the mixture was determined and then the samples were formed into molds. After shaping and demolding, the samples were stored in a bathtub over water at 20 °C and RH ≥ 95% until the bending strength and compressive strength were determined. In the case of other tests, the samples were additionally seasoned in accordance with the requirements of individual test standards performed as part of the described experiment.

### 2.3. Methodology

Initial W/C ratio of 0.5 was assumed, similar to the standard cement mortar. This ratio was used to prepare the MR reference mortar. Due to the porous structure of the ceramic waste aggregate, additional water had to be added during mixing in order to obtain a comparable consistency of all analyzed mortars. Consistency was determined with the flow table method according to EN 1015-3. Additional water was added experimentally. Its total amount, together with the batch water, is shown in Table 1.

The pressure method (1 L volume of mix) in accordance with EN 1015-7 (Method A) was used to determine the air content. For compressive strength and flexural strength determination after 7, 28 and 56 days of maturation, samples were prepared according to EN 1015-11 (40 × 40 × 160 mm prisms). Determination of capillary action was performed in accordance with EN 1015-18 (40 × 40 × 160 mm prisms). The study also determined the heat transfer coefficient (100 × 100 × 50 mm) using the nonstationary method. Samples of dimensions 100 × 100 × 10 mm were used for sorption measurements. The samples after demolding were cured over water at 20 °C and ambient relative humidity RH > 95% until tests.

Before starting the actual sorption tests, the samples had to be first wet cut with a diamond saw into 10 mm slices. This allowed us to achieve samples with following dimensions length 100 mm, height 100 mm and thickness 10 mm. The next step was to insulate the edges of the samples with silicone. The samples were oven-dried at 70 °C until they reached a constant mass.

#### 2.3.1. Hygroscopic Sorption Measurements Using Gravimetric Method

The measurement of sorptivity began when the samples were placed in an airtight container with a set relative humidity. For this purpose, saturated salt solutions were used (Table 2). Samples were placed on grids and locked in containers with relative humidity of RH ≈ 11%, 33%, 59%, 75%, 85%, 97% respectively. The containers were placed in a climate chamber that maintained a constant temperature of 20 °C.

The sorption tests were performed by recording the mass of the samples, which changed due to different ambient conditions. The tests were conducted until the mass of the samples stabilized, which was when the change in mass between two successive measurements was not greater than 0.1%, as recommended in ISO 12571.

#### 2.3.2. Sorption Measurement with a DVS Method

Sorption measurements were also made using the DVS method. Dynamic Vapor Sorption (DVS) is one of the leading gravimetric methods used to study the sorption or desorption of water vapor. The tests were performed on an “IntrinsicPLUS” apparatus. Twenty-four relative humidity levels were programed from 0 to 97% (0% to 95% every 5% and 97%).

## 3. Results and Discussion

### 3.1. Fresh Mortar Consistency According to the Standard EN 1015-3

**Figure 3:**
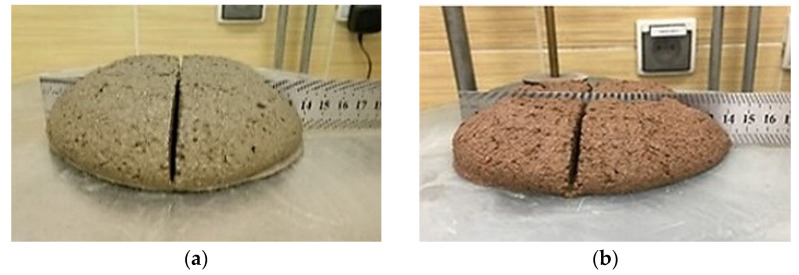
Measurements of consistency in a slump flow test. (**a**) reference mix; (**b**) mix with recycled ceramic waste M50.

Table 3 presents the results of consistency measurements of fresh mixtures as determined on a flow table. The mixes were classified in accordance with EN 1015-3. The assumed slump flow was in the range of 140–200 mm. Figure 3 presents consistency measurements of mortars.

The additional water necessary due to porous structure of waste aggregate was precisely added to the mix. Similar results were obtained for all of the mixes, which allows for easier correlation of other results, due to exclusion of consistency factor.

Lack of water halo in the flow table tests of the M50 mortar (Figure 3), means that the water is entrapped within the grains of waste aggregate even when vibrations are introduced.

The recycled ceramic waste is characterized by a porous structure with up to approx. 20% of pore volume. Importantly, the additional water is not bound by the initial hydration [1,11,30]. It will evaporate from the mortar over time. However, the water absorbed by the grains of recycled ceramic waste will be retained in the structure for a long time, allowing for self-curing of the cement matrix to happen.

### 3.2. Air Content in Fresh Mortar According to the Standard EN 1015-7

After obtaining proper mortar consistency, samples were tested for air content (Method A). The results of the air content in mixtures are presented in Table 4.

**Figure 4:**
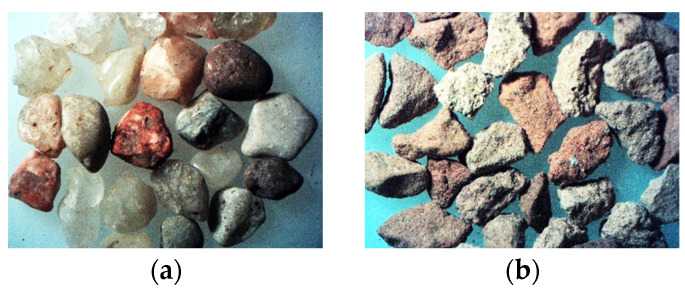
Aggregate grains of the 1.6/2.0 fraction (10×) (**a**) natural sand; (**b**) recycled ceramic waste.

It was clearly visible that the type of aggregate has a significant influence on the compaction of the mixes. It should be mentioned that the natural aggregate grains are oval, while the ceramic grains are elongated and have a rougher surface (Figure 4). This is caused by the grinding process of the ceramic rubble and the porous structure of the recycled ceramic. Therefore, the dispersion and positioning of the grains is variable and does not always guarantee a proper compaction.

### 3.3. Dry Bulk Density in Accordance with EN 1015-10

Determination of the dry bulk density of mortars was performed in accordance with EN 1015-10. The test was performed on samples 100 × 100 × 50 mm. Mean dry bulk density obtained in the test was presented in Table 5.

Highest dry bulk density was obtained for the reference mix, while the lowest was obtained for mortar with the highest content of the RCA. This is caused by the porous structure of the recycled aggregate.

### 3.4. Determination of the Flexural Strength of the Hardened Mortar According to the Standard EN 1015-11

Determination of the flexural strength was conducted in accordance with EN 1015-11 after 7, 28 and 56 days of curing. Results are presented in Table 6.

**Figure 5:**
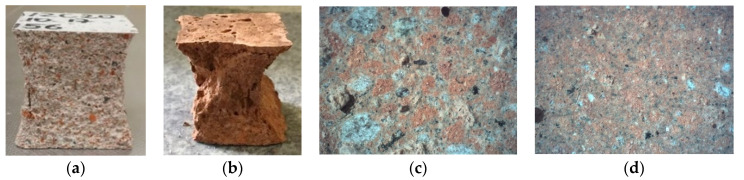
(**a**,**b**) Determination of flexural strength of mortars with recycled aggregate: The M20 and M100 samples after compressive strength determination test (28 days); (**c**) image of the M50 mortar structure at a magnification of 20×; (**d**) image of the M100 mortar structure at a magnification of 20×.

Results of tests after 7 days of curing showed lower flexural strength of mortars with waste aggregate in relation to the reference mortar. Tests carried out after 28 and 56 days of curing showed an increase of strength with time for given mortars. However, there was no visible correlation between the amount of recycled aggregate and the flexural strength. It is visible in Figure 5a that, in the sample after determination of the compressive strength, there are individual grains of ceramic aggregate with larger grain size, which can influence the uneven transfer of bending stresses. Furthermore, the failure mode of the specimen clearly indicates the fracture of ceramic grains. Surprisingly, the results of the specimens with 100% RCA after 56 days of curing where comparable to the reference mortar. It is worth noting that the ceramic grains have a porous structure and a rough surface, which promotes good cooperation in the Interfacial Transition Zone (ITZ). This effect is enhanced by the migration of the cement paste deep into the open pores (Figure 5c,d). In addition, the water absorbed by the porous ceramic grains during the mixing of the components improves the internal self-curing [31].

### 3.5. Determination of the Compressive Strength of the Hardened Mortar According to the Standard EN 1015-11

The determination of compressive strength was performed according to PN-EN 1015:11. Samples for compressive strength testing were taken from halves of prisms used in the flexural strength determination. The test was carried out on six samples of each mortar. The force was applied axially to the specimen surface. The samples were compressed with a hydraulic press until failure (Figure 5a,b). Table 7 shows the results of the compressive strength determination.

The use of recycled ceramic aggregates reduces the compressive strength of mortar in relation to the reference one. The lowest values of the compressive strength were determined for the mortar with 100% of RCA. The ceramic aggregate grains have lower strength than natural sand. Even though the use of RCA reduces the strength of mortars, the results obtained in the study still allow us to classify them as masonry mortars with the Md class. The compressive strength after 28 days was in all studied samples higher than 25 MPa (EN 998-2).

### 3.6. Water Absorption Due to Capillary Action According to the Standard EN 1015-18

**Figure 6:**
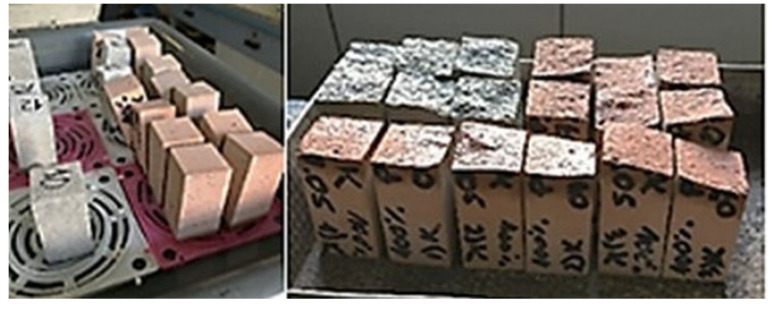
Determination of the water absorption due to capillary action.

Determination of the water absorption coefficient due to capillary action was carried out in accordance with PN-EN 1015-18 (Figure 6). For this purpose, specimens with dimensions of 40 × 40 × 160 mm were used, broken into halves, similarly to procedures in flexural strength test. The halves of specimen were then dried at 70 °C to a constant weight. Table 8 presents the mean values of water absorption coefficient due to capillary action of studied mortars.

Results of the water absorption coefficient due to the capillary action showed that the RCA increased the value of this coefficient. The higher the RCA content, the higher the increase. Water absorption is directly correlated to the porosity material. Capillary forces depend primarily on the number of open pores and their diameter. Materials with a higher level of capillary action are characterized by a large number of microscopic open pores. RCA has significantly more open pores than natural sand. Adding it to the mortar increases the capillary action, which means the material is able to pull more water into the air voids (pores). This has a negative effect on the properties of the mortar, e.g., freeze/thaw resistance and strength of the material. The use of different binders, reduction of water–cement ratio, use of mineral additives and chemical admixtures reduce the penetration of water into the material [32].

### 3.7. Water Absorption

Water absorption was determined by soaking. The test results showed how much water can be absorbed by the specimen placed under water. Results are presented in Table 9.

Both results of capillary action and water absorption determination show that samples with recycled aggregate have higher results. The porous structure of the aggregate allows for more water to be absorbed.

### 3.8. Thermal Conductivity Coefficient

The thermal conductivity coefficient of the hardened mortar was determined using a nonstationary method with an ISOMET 2104 [33]. Table 10 presents the mean values of thermal conductivity coefficient of the samples in dry state.

The thermal conductivity coefficient decreases with the amount of RCA in mortars. The highest thermal conductivity coefficient was determined for the M100 mortar with 100% of RCA. Better thermal characteristics of the RCA are caused by its porous structure. The lower the thermal conductivity coefficient the better insulator the material is [33]. This means that there is a lower risk of thermal bridges where the mortar interacts with masonry.

### 3.9. Moisture Sorption

**Figure 7:**
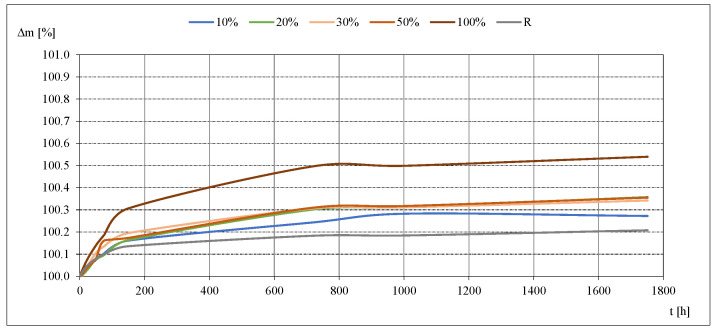
Moisture sorption kinetics at RH = 33%.

**Figure 8:**
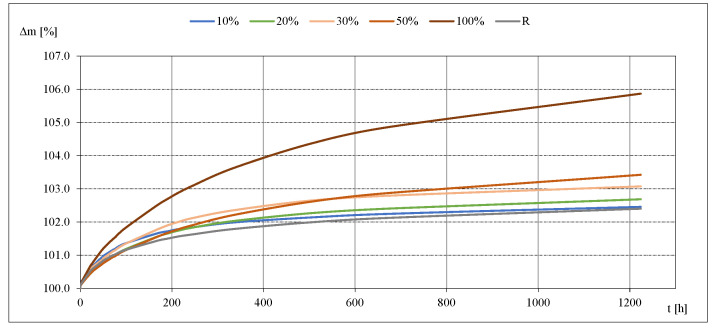
Moisture sorption kinetics at RH = 85%.

Results of the determination of moisture sorption of studied mortars are presented in Table 11. Kinetics of moisture sorption for studied mortars at 33% and 85% of RH are presented in Figure 7 and Figure 8.

#### 3.9.1. Results of Sorption Determined with Gravimetric Method

From the results obtained, it was possible to determine the equilibrium sorption moisture content *w* (%) (Table 11), as a percentage of the moisture content in relation to the dry mass.

**Figure 9:**
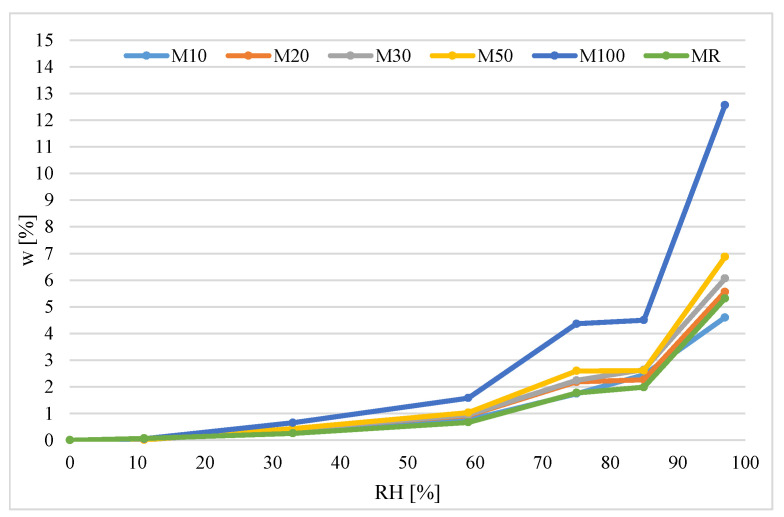
Sorption isotherms of the tested materials.

The moisture sorption *w* (%) created the sorption isotherms for mortars with different RCA content. The isotherms are presented in Figure 9.

#### 3.9.2. Sorption Measured Using DVS Method

Equilibrium sorption moisture content *w* (%) measured with the DVS method has been presented in Table 12.

**Figure 10:**
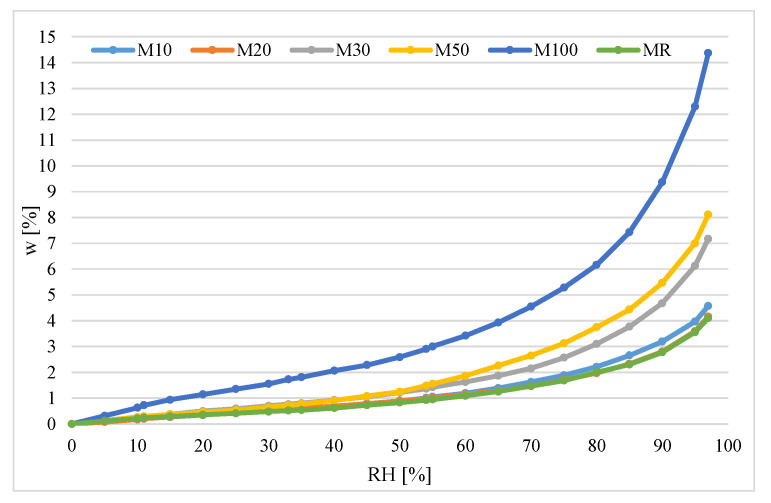
Sorption isotherms for studied material obtained from DVS methods.

The results presented in Table 12 created the sorption isotherms for mortars with different RCA content. The isotherms are presented in Figure 10.

#### 3.9.3. Kinetics of the Sorption Processes in Mortars with RCA Content

Based on the detailed data collected during the measurements, the kinetics of sorption process were evaluated for the studied mortars. Exemplary graphical representations (Figure 7 and Figure 8) show that the most intensive development of the sorption kinetics process took place in the first phase of measurements, i.e., during the first several dozen hours of the study. As the process continued, the mass changes became smaller. The moisture equilibrium was reached faster at the lowest moisture contents, i.e., at 11 and 33%. It took the longest time at the highest relative air humidity. The process was most intensive for mortar with 100% RCA. 

The nature of the determined curves is related to the formation of a multimolecular adsorption layer during physical adsorption [34]. In the reconstructed curves, three parts can be distinguished corresponding to the three stages of gas adsorption on the solid. At low relative humidity, a monomolecular layer of the adsorbed substance forms on the surface of the adsorbent. Then, as the relative air humidity increases, the formation of a multimolecular layer takes place. Above the humidity of about 80%, the capillary condensation process begins in the adsorbent mesopores. Visible differences in the value of sorption moisture of the tested materials and the course of the process are caused by the different specific surfaces of the tested materials and the different porosity structures.

#### 3.9.4. Evaluation of the Sorption Equilibrium of Mortars with Recycled Ceramic Aggregate

Highest moisture sorption was achieved at higher ambient relative humidity. This result is concurred by other studies [35,36,37].

In the traditional method, the highest moisture content of 12.6% was obtained for mortar samples with 100% RCA at 97% RH. In the same method, at a relative humidity of 11%, all materials tested showed negligible moisture absorption (<0.1%). In the case of the DVS method at 11% humidity, the degree of moisture absorption by the material was higher and amounted to 0.2–0.3. In the case of material with 100% RCA, the result was an even 0.7%.

According to the data, ceramic products are among the least hygroscopic materials [38]. Ceramic brick is characterized by very low sorptivity, not exceeding 1.5%. Lime and sand products absorb about 10 times more than clay brick. The lowest moisture sorption among building materials is that of ceramics, ordinary concrete, mineral wool, gypsum concrete, and it does not exceed 3% by weight. Lime sand brick, cement mortar and cellular concrete have a moisture sorption of several percent.

The sample containing 100% RCA achieved the highest value of moisture sorption among all tested mortars. Comparing the results to the literature data for ceramics, it can be observed that the obtained values disprove the statement that ceramic is the material with the lowest hygroscopicity. Replacement of the natural sand with recycled material probably contributed to a change in the structure and increased its porosity. However, this statement requires further study. A similar situation was observed for the results from the DVS method. In this case, for a sample containing 100% RCA, a moisture sorption value of 14.4% was obtained at RH = 97%. This value is similar to the one obtained with traditional methods. The low sorption capacity of ceramics is confirmed by the research presented in [39]. There, for ceramic bricks, the sorption moisture was 1.9% at 5 °C, 1.7% at 20 °C and 1.5% at 35 °C. The tests were performed using the gravimetric method.

The difference of the results obtained in the DVS method for RH = 11% from results obtained with a traditional method reached up to 0.7% for mortar with 100% RCA.

Regardless of the method used, with the increase of the relative humidity, results of moisture sorption also increased. Some studies, however, do not concur with this result [35,36,40].

**Figure 11:**
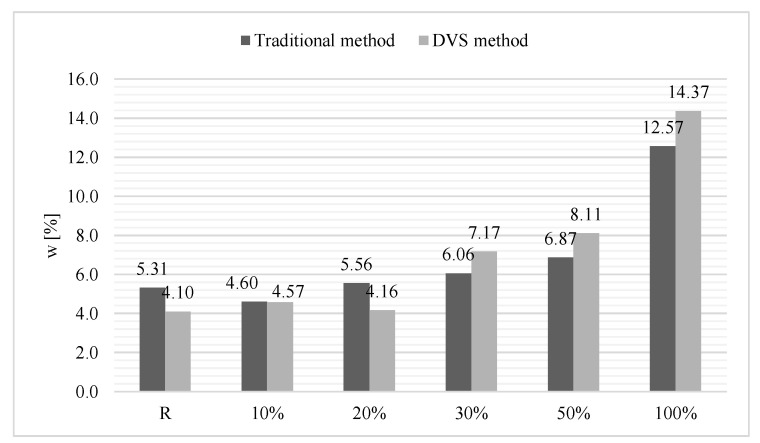
Moisture sorption at RH = 97%.

The influence of the RCA content in mortars on the moisture sorption was evaluated based on the values obtained at RH = 97%. Results are presented in Figure 11. It can be concluded that the replacement of 100% natural aggregate with recycled ceramic aggregate negatively affects the absorption of moisture from the environment, causing a more than twofold increase in sorptivity compared to the reference mortar in the traditional method and more than threefold increase in the DVS method. The addition of recycled ceramic aggregate in the amounts of 10 and 20% does not significantly affect the sorptivity. However, increases of RCA content to 30% and 50% result in increased moisture absorption by the mortars.

#### 3.9.5. Evaluation of the Sorption Isotherms for Mortars with RCA

Results obtained in this study (Figure 9 and Figure 10) have shown that the isotherms for the mortars with RCA are of the same type. They can be classified both according to Brunauer as well as IUPAC as type 2 isotherms. Looking at the isotherms for the RH = 75–85%, a characteristic inflection point is visible. This points the transition to the next sorption phase, where capillary condensation takes place, which causes sudden development of sorption.

In the case of the traditional method, we can observe on the graph that samples with 10%, 20%, 30% and 50% of RCA, as well as reference samples, obtained very similar sorption isotherms. The isotherm of the sample with 100% RCA differs significantly from them. This is also visible for where the DVS method is applied.

Results presented in Figure 9 and Figure 10 show that the DVS method provides more results in a shorter time, and the plotted isotherms are more accurate than in the traditional method. In the traditional method, there were only 6 levels of relative humidity, while in DVS method there were 24 levels.

## 4. Conclusions

The results of the experimental studies can be summarized as follows:

1. An increase in the amount of RCA resulted in a decrease in the density of the cement mortar. This is due to the porous structure of the grains of ceramic waste.
2. The proportions of natural aggregate and recycled ceramic aggregate in the mix affected the compaction rate of individual mixes and their air content.
3. The angular shape of the grains made of the recycled waste influenced the workability of the mix due to blocking effect. In mixes with mixed aggregate shapes, the compaction was better, limiting the porosity of the interfacial transition zone.
4. A lower flexural strength was observed for mortars with RCA compared to the reference mortar. Surprisingly, the results carried out on samples with 100% RCA showed that flexural strength after 56 days of maturation was comparable to the reference mortar. The effect was concluded to be a result of stronger interfacial transition zone due to higher specific surface area of recycled grains and better paste penetration into open pores.
5. The addition of recycled ceramic aggregate reduced the compression, regardless of the testing age to the reference mortar. The lowest compressive strength values were obtained for mortar with 100% RCA. This is a result of porous structure of recycled aggregate, so different from natural sand.
6. The RCA increased the value of capillary adsorption coefficient. The samples with the highest RCA content obtained the lowest values.
7. The RCA increased the number of pores in the mortar and thus caused increased water absorption.
8. An increase in the content of RCA in the mortar lowered the thermal conductivity coefficient.
9. As expected, all tested samples showed insignificant moisture sorption. The sorption isotherms, which were obtained for the studied materials, represent type II isotherms according to Brunauer classification and type II according to IUPAC classification.
10. A strong correlation was found between the amount of RCA and moisture sorption. The reference mortar and mortars with 10%, 20% and 30% of RCA absorbed the least moisture. However, in both measurement methods, at each level of relative humidity at 20 °C, the highest value of moisture sorption was obtained for the sample with 100% RCA.
11. Replacing 100% natural aggregate with 100% recycled ceramic aggregate negatively affected the absorption of moisture, resulting in increased sorptivity compared to the reference mortar.
12. Analyses also showed that testing with both methods simultaneously provides confidence in the reliability of the results obtained and gives the possibility of a quick assessment of the isotherms obtained and the possible need for repetition of the study.

The study on composites with recycled ceramic aggregate can be a starting point for further research on these types of materials and fits into the notion of sustainable development because the obtained results show a potential of using these materials in construction.

In conclusion, it should be stressed that the results obtained in this study and the sorption isotherms of cement mortars with recycled ceramic aggregate improve the current knowledge and can become useful in the case of simulating the thermal–humidity processes of those materials.

## Figures and Tables

**Table 1:** Total water amount in prepared mixes.

Mix	MR	M10	M20	M30	M50	M100
Total W/C Batch water + added water	0.5	0.55	0.61	0.66	0.91	1.28

**Table 2:** Relative humidity in containers with salt solutions.

Temperature	Relative Humidity RH (%)					
	Lithium Chloride LiCl	Magnesium Chloride MgCl_2_	Magnesium Nitrate Mg(NO_3_)_2_	Sodium Chloride NaCl	Potassium Chloride KCl	Potassium Sulfate K_2_SO_4_
20 °C	11.31 ± 0.31	33.07 ± 0.18	59.14 ± 0.44	75.47 ± 0.14	85.11 ± 0.29	97.59 ± 0.53

**Table 3:** Slump flow in accordance with EN-1015-3.

Mix	MR	M10	M20	M30	M50	M100
Slump flow (mm)	141	140	140	140	142	145

**Table 4:** Air content in studied mixes in accordance with EN-1015-7.

Mix	MR	M10	M20	M30	M50	M100
Air content (%)	7.0	8.8	9	9.5	7.4	6.4

**Table 5:** Dry bulk density in accordance with EN-1015-10.

Mix	MR	M10	M20	M30	M50	M100
Dry bulk density (g/cm^3^)	2.15	2.11	2.05	2.20	1.92	1.81

**Table 6:** Flexural strength of mortar in accordance with EN-1015-11.

Mortars	MR	M10	M20	M30	M50	M100
Age (Days)	Flexural Strength (N/mm^2^)					
7	6.19	5.87	5.45	5.15	4.98	4.44
28	6.22	6.06	6.41	6.22	6.68	6.08
56	7.20	7.07	7.07	6.47	6.58	7.25

**Table 7:** Determination of compressive strength in accordance with EN-1015-11.

Mortar	MR	M10	M20	M30	M50	M100
Age (Days)	Compressive Strength (N/mm^2^)					
7	49.49	43.86	37.64	35.29	30.88	23.38
28	50.21	45.94	42.23	37.05	36.50	30.87
56	55.23	48.54	48.12	42.45	37.67	34.77

**Table 8:** Water absorption due to capillary action according to EN-1015-18.

Mortar	MR	M10	M20	M30	M50	M100
Mean value (kg/m^2^·min^0.5^)	0.16	0.16	0.18	0.20	0.30	0.70

**Table 9:** Results of water absorption conducted in accordance with EN-1015-18.

Mortar	MR	M10	M20	M30	M50	M100
Mean value (%)	6.12	6.61	7.82	9.03	13.22	19.88

**Table 10:** Mean values of thermal conductivity coefficient of studied mortars.

Mortar	MR	M10	M20	M30	M50	M100
	Mean Value (W/m∙K)					
Dry state	1.99	1.89	1.59	1.37	1.13	0.68

**Table 11:** Mean equilibrium moisture sorption *w* (%).

Relative Humidity *RH* (%)	Sample					
	MR	M10	M20	M30	M50	M100
11	0.066	0.035	0.023	0.007	0.013	0.054
33	0.258	0.349	0.424	0.403	0.435	0.649
59	0.664	0.759	0.920	0.951	1.032	1.578
75	1.769	1.739	2.184	2.241	2.596	4.360
85	1.985	2.451	2.262	2.641	2.608	4.500
97	5.314	4.601	5.560	6.059	6.872	12.566

**Table 12:** Mean sorption results *w* (%) obtained with DVS method.

Relative Humidity *RH* (%)	Sample					
	MR	M10	M20	M30	M50	M100
0	0	0	0	0	0	0
5	0.121	0.076	0.074	0.133	0.140	0.315
10	0.213	0.174	0.177	0.267	0.268	0.626
11	0.234	0.201	0.204	0.294	0.290	0.723
15	0.293	0.269	0.279	0.377	0.367	0.936
20	0.355	0.347	0.370	0.497	0.456	1.145
25	0.412	0.423	0.442	0.590	0.536	1.353
30	0.478	0.496	0.517	0.697	0.643	1.557
33	0.520	0.548	0.572	0.764	0.720	1.727
35	0.543	0.587	0.605	0.804	0.772	1.814
40	0.617	0.673	0.696	0.921	0.908	2.067
45	0.728	0.778	0.791	1.058	1.080	2.280
50	0.826	0.897	0.893	1.214	1.242	2.590
54	0.923	1.010	0.979	1.361	1.481	2.901
55	0.954	1.050	1.013	1.425	1.554	3.003
60	1.093	1.193	1.140	1.625	1.866	3.420
65	1.255	1.388	1.286	1.868	2.256	3.929
70	1.466	1.626	1.469	2.152	2.649	4.550
75	1.679	1.883	1.696	2.569	3.123	5.273
80	2.006	2.211	1.971	3.099	3.744	6.166
85	2.307	2.655	2.328	3.766	4.435	7.432
90	2.775	3.189	2.802	4.677	5.467	9.367
95	3.557	3.964	3.592	6.125	7.003	12.297
97	4.102	4.574	4.162	7.173	8.105	14.369

## Data Availability

The data presented in this study are available upon reasonable request from the corresponding author.
